# A Study on Microturning with Electrochemical Assistance of the Cutting Process

**DOI:** 10.3390/mi9070357

**Published:** 2018-07-19

**Authors:** Marcin Grabowski, Sebastian Skoczypiec, Dominik Wyszynski

**Affiliations:** Institute of Production Engineering, Cracow University of Technology, al. Jana Pawla II 37, 31-864 Krakow, Poland; skoczypiec@mech.pk.edu.pl (S.S.); wyszynski@mech.pk.edu.pl (D.W.)

**Keywords:** assistance passivation, electrochemical, hybrid, microcutting, micromachining, microturning, precision machining

## Abstract

The paper investigated an electrochemically-assisted microturning process. Depending on the variant of electrochemical assistance, material can be removed with simultaneous electrochemical and mechanical action or electrochemical assistance can change the conditions of the cutting by changing the mechanical properties of the machined material. The experimental part includes discussion of the study methodology and a comparison of straight turning results in the case of machining 1.4301 stainless steel with and without electrochemical assistance. Based on this study, we can confirm that electrochemical assistance brings significant benefits in both variants, especially when the depth-of-cut is in the range of 1 µm.

## 1. Introduction

Scaling down the turning process determines size effects and changes of the process itself, including behavior and machining characteristics [1]. Decreasing the depth-of-cut thickness makes that relation between the uncut chip thickness and the cutting forces become nonlinear and causes specific cutting energy increases (more energy is required to remove material). The intensity of a size effect depends on the machined material’s properties (stress and strain) and other factors (e.g., frictional effects and cutting edge conditions). Unfortunately, the occurrence of a size effect dictates that the microturning process is suitable only for shaping parts made of soft materials (i.e., plastics, brass, mild steel). 

The negative impact of the size effect can be overcome by using an additional energy source in the machining area [2,3]. During such a process, the role of additional energy is to improve the machined material’s machinability or to modify the mechanism of material removal. An example of a practical implementation of the first strategy is thermal-assisted machining [4], while the ultrasonic vibration-assisted cutting process is the example of the second approach [5].

In laser assisted machining (LAM), a laser heats the workpiece in front of the cutting tool which leads to a reduction in strength and to a higher material removal rate [6,7,8]. Laser assistance in micromachining is mainly used in turning operations where the process kinematics help to keep constant parameters of laser influence. Properly designed laser-assisted microturning improves process efficiency and reduces cutting tool wear, which results in improved quality of the surface layer [9,10].

The application of tool ultrasonic vibrations [11,12,13,14,15] is another efficient solution of microturning, drilling, grinding, or milling process assistance. In vibration-assisted machining (VAM), the shape of the chips is changed and the tool built-up edge is reduced, allowing one to minimize the temperature in the cutting zone as well as to reduce cutting forces, therefore reducing tool wear [16,17]. A relatively new approach to process assistance in microcutting is the use of cryogenic cooling, where tool wear is reduced due to a decrease in temperature in the cutting zone and due to change in the mechanical properties of the cutting layer. Such a solution is particularly effective when machining ductile materials [18,19]. 

It has to be emphasized that the above-mentioned solutions are not easy in the case of micromachining. In the LAM process, the problem with effective heat dissipation occurs, while in the VAM process, tool or part vibration can negatively influence machining accuracy. Therefore, it is worthwhile to consider electrochemical assistance (ECA) in micromachining because it does not introduce additional forces or heat to the machining area. Depending on its parameters, electrochemical assistance can change the mechanical properties of the machined material through passivation, or material can be removed with simultaneous electrochemical and mechanical action.

The idea of combining electrochemical and mechanical interaction during removal of material was developed in the 1960s as the electrochemical grinding (AECG) process. In AECG simultaneous, mechanical and electrochemical material removal allows one to improve the quality of the surface layer, thus increasing productivity, while tool wear and energy consumption decrease. AECG is effective for machining parts made of difficult-to-cut materials, such as sintered carbides, titanium alloys, nickel alloys, and metal matrix composites [20]. Zhu, et al. [21] presented an example of successful application of AECG in the micromachining domain. In the process that they developed, a metal rod with coated abrasives used as a cathode tool rotates at a high speed and removes the material electrochemically and mechanically for a pre-machined pilot hole. In this process, the settings of the main machining parameters (voltage, cathode rotation speed, and feed rate) determine the method of material removal, which can be balanced between mechanical and electrochemical.

The Pourbaix diagram implies that for certain conditions of the electrochemical process (called as passive state), a thin electrolytic oxide layer grows on the workpiece surface. This was used in the electrolytic in-process dressing (ELID) technology [22]. In ELID, grinding electrolytic action takes place between the metal bonded grinding wheel (anode) and a conductive cathode. The anodic oxide layer grows from the metal bond. This layer wears off easily during grinding and exposes sharp abrasive grains and thus performs efficient grinding action. The application of the same effect to improve efficiency of the micromilling process was proposed in Ref. [23]. The study consists of passivation of the workpiece surface and then cutting of a 1-µm thick layer of material. When machining a non-passivated surface after five passes, it was possible to remove only 1 µm of machining allowance due to a ploughing effect, but when machining a passivated surface with the same cutting parameters, 7 µm of allowance thickness was removed instead. In Ref. [24], the authors state that this effect correlates with the thickness of a passive film. In Ref. [25], the ellipsometry technique, optical profilometry, and nanoindentation tests were applied to determine properties of the 1.4301 steel’s passivated surface. In this research, the oxide layer thickness did not exceed 100 nm. Surface topography analysis (roughness distribution and Fourier transforms) after passivation showed that the surface state changed from an anisotropic to an isotropic one. A significant difference in the microhardness of the surface layer before and after passivation was also observed. This led to the conclusion that the oxide layer created on the machined surface is harder than the core material.

In the following paragraphs, the possibilities of ECA in the microturning process will be discussed and experimentally verified.

## 2. Materials and Methods 

The concept of ECA in the microturning process was verified in straight turning kinematics. The reason for choosing straight turning was that, in this case, electrochemical assistance and the cutting process can take place simultaneously. The two following variants were investigated:

- Variant *A*, in which the parameters of ECA are typical for the electrochemical grinding process (in this case, simultaneous mechanical and electrochemical material removal take place). Variant *A* allows combining the advantages of mechanical (high accuracy) and electrochemical microturning (no mechanical forces during machining, no tool wear) and to simultaneously reduce the disadvantages of both processes (in mechanical microturning it is a relatively high specific cutting energy and in electrochemical microturning it is the relatively low accuracy).
- Variant *B*, in which the role of ECA is only to change the properties of the machined material. In variant *B*, the change in material removal conditions may occur, therefore a decrease of both the cutting force and tool wear is expected.

We selected 1.4301 stainless steel as the machined material. It is noteworthy that 1.4301 steel belongs to group of materials which could be passivated, therefore an outer layer of iron oxide can be created on the work surface. It belongs to a group of the most common and therefore most frequently machined austenitic stainless steels. Machining of 1.4301 steel is very challenging due to its high ductility, significant work hardening rates, tendency to built-up edges on the cutting tool, and poor chip breaking properties. These are particularly relevant when the cutting depth is in the range of minimal chip thickness and the ploughing effect plays a significant role. Such conditions occur during the microturning process. For both variants of assistance, the research was carried out on the test stand described in detail in Ref. [26]. The tests were performed in accordance with the experiment theory with the input and constant parameters as in Table 1.

For variant *B*, a 1 µm depth of cut *a_p_* was assumed, which is in agreement with conclusions from Ref. [25], where the authors established that there is no justification for utilizing electrochemical assistance for higher than 1 µm values of *a_p_*. It is significant that the value of the shaft diameter was far from the micromachining domain, however, the assumed value was intentional as to facilitate force measurement. On the other hand, however, the assumed cutting depth was within the microcutting range. Each test dealt with removing *n* material layers without ECA and then, on the same shaft, the removal of another *n* number of material layers was performed by application of ECA. Each test was carried out by applying a new cutting tool. During the tests, the signal of the force was consecutively registered, smoothened, and then analyzed. The difference *ΔF* defined in the following way was selected and served as the main output parameter:

(1) $$\Delta F=\bar{F}-{\bar{F}}_{ECA}$$

where: $\bar{F}$ means cutting force during normal (unassisted) turning and ${\bar{F}}_{ECA}$ means cutting force during electrochemically assisted turning. The average values were calculated from *n* tool passes (*n* = 3 for variant *A* and *n* = 10 for variant *B*). Additionally, the relations of force load *F* and *ΔF* from the investigated parameters were approximated assuming the full quadratic polynomial (constant, linear, interaction, and squared terms) as a response surface. For adequate choice of the electrochemical processing, Pourbaix diagrams have been analyzed.

## 3. Results and Discussion

**Figure 1:**
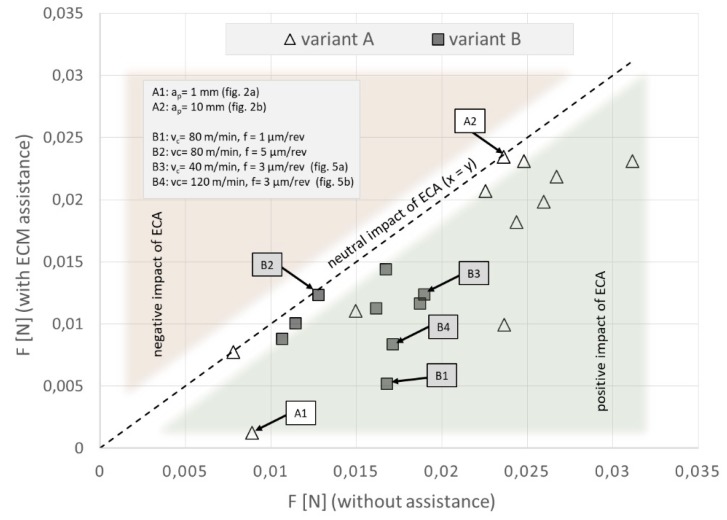
Correlogram presenting the relationship between force sensor load *F* with and without electrochemical assistance (ECA) for investigated variants of machining.

Results of whole tests are presented collectively in Figure 1. The average cutting forces were in the range of millinewtons and machining with ECA led to reduction of sensor load *F*, regardless of investigated variant (*A* or *B*).

**Figure 2:**
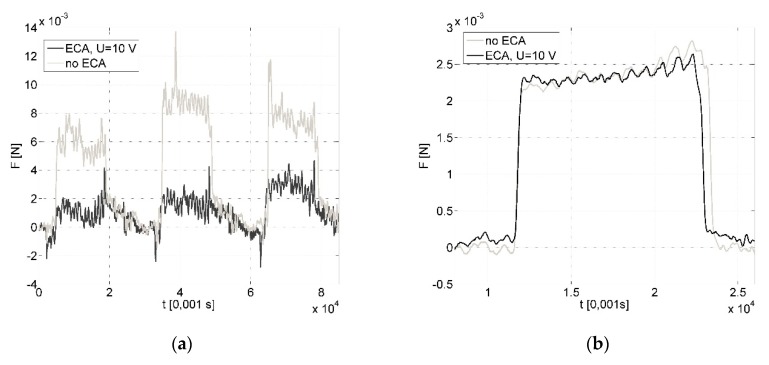
Relation of force sensor load during cutting with the following parameters: feed rate *f* = 3 µm/rev, cutting speed: *V_c_* = 80 m/min; depth of cut *a_p_* = 1 µm (**a**) and *a_p_* = 10 µm (**b**), ECA in variant *A*.

In variant *A*, for the examined cutting parameters’ range, ECA brought the most benefits for *a_p_* = 1 µm. While for higher values of *a_p_*, *ΔF* is at the measurement error level (Figure 2). Further analysis which consists of an approximation of the relations is presented below.

**Figure 3:**
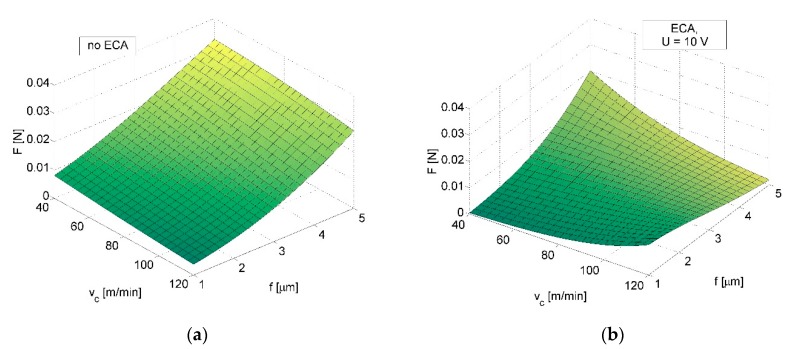
Relationship between sensor load and cutting speed *V_c_* and feed rate *f* for *a_p_* = 3 µm; machining without ECA (**a**) and machining with ECA in variant *A* (**b**).

**Figure 4:**
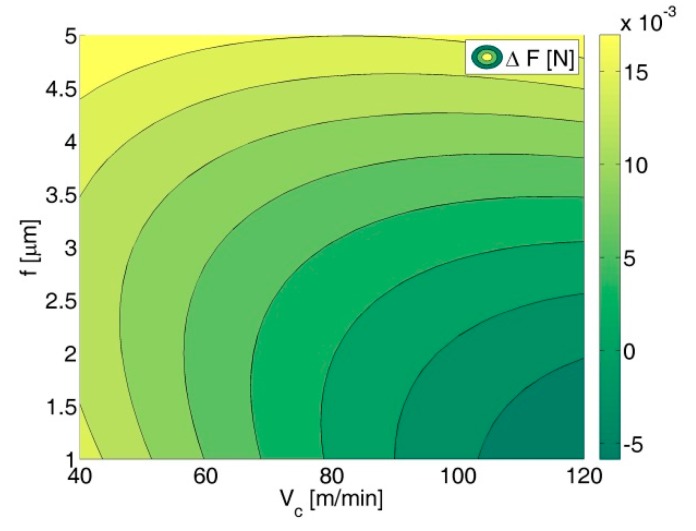
Contour map of the sensor load decrease *ΔF* due to ECA in variant *A* (relation between *ΔF*, cutting speed *V_c_,* and feed rate *f*, *a_p_* = 3 µm).

*F* (*V_c_*, *f*, *a_p_*) and *ΔF* (*V_c_*, *f*, *a_p_*) indicated the reduction of force load resulting from ECA (Figure 3a vs. Figure 3b) and led us to the conclusion that ECA is most beneficial for *a_p_* = 5 µm and depends on *V_c_* and *f* values (Figure 4). It is noteworthy that for *V_c_* = 100 m/min, *f* = 2 µm/rev and *a_p_* = 3 µm relation *ΔF* (*V_c_*, *f*) has negative values. However, within this range, the measurements were affected by the largest errors.

Measured with the Taylor Hobson Surtronic roughness tester (Form Talysurf i-Series profilometer, AMETEK Inc, Berwyn, PA, USA), the values of surface roughness were:

- *Ra* in range 0.10−0.43 µm and *Rz* in range of 1.20−2.25 µm when machining was carried out without ECA,
- Almost similar for all samples machined with variant *A* (*Ra* = 0.08−0.15 µm and *Rz* = 0.5−1.15 µm, standard deviation *σ_Ra_* = 0.02 μm and *σ_Rz_* = 0.22 μm).

The same parameters of ECA were used for all tests, and in each experiment, the current was ≈ 0.5 A which gives a current density ≈ 10 A/cm^2^. In electrochemical machining, this value is usually considered a lower limit for effective anodic dissolution. Therefore, it can be concluded that in variant *A*, phenomena related to electrochemical machining had the dominant influence on surface layer quality. 

**Figure 5:**
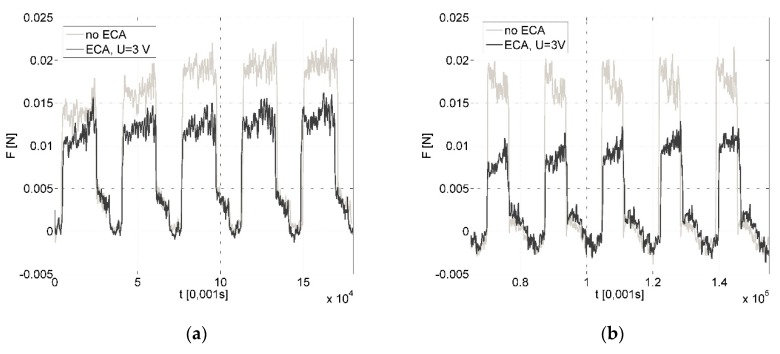
Relation of force sensor load during cutting with the following parameters: feed rate *f* = 3 µm/rev, cutting speed: *V_c_* = 40 m/min (**a**) and *V_c_* = 120 m/min (**b**), ECA in variant *B*.

**Figure 6:**
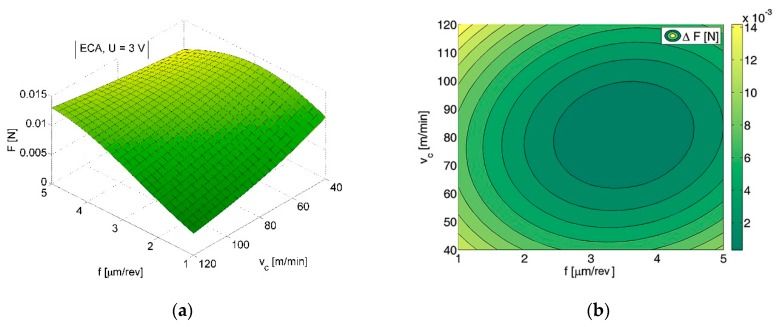
Relationship between (**a**) the sensor load and cutting speed *V_c_* and feed rate *f*; (**b**) contour map of the sensor load decrease *ΔF* (relation between *ΔF*, cutting speed *V_c_*, and feed rate *f*); machining with ECA in variant *B*.

In variant *B*, ECA also plays a significant role for reduction of the sensor load during machining (Figure 5). The average cutting force decreases (*ΔF*) from a few to several millinewtons, which gives a percentage reduction from 5% to 65% in comparison to the force value when machining without ECA. The value of the sensor load depends on the feed rate *f* and cutting speed *V_c_*, and the values most beneficial for ECA are *V_c_* = 120 mm/min and *f* = 1 µm/rev (Figure 1). The influence of assistance becomes unimportant [2] with the feed increases (Figure 6). Due to the decrease in feed, the chip thickness gets smaller and the force increases (for *f* = 1 µm/rev, *F* = 0.0168 N vs. *F* = 0.0128 N for *f* = 5 µm/rev) and tool working time gets longer. It can be asserted that ECA in variant *B* is beneficial when the undeformed chip thickness remains in the passive layer thickness range.

**Figure 7:**
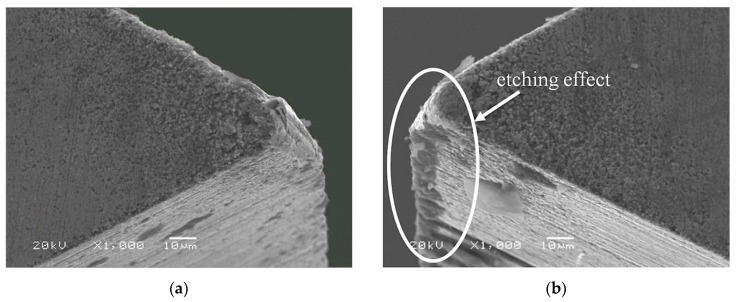
SEM photographs of the cutting edge after removing 3 layers of material with the following cutting parameters: depth of cut *a_p_* = 6 µm, cutting speed *V_c_* = 80 m/min, feed rate *f* = 3 µm/rev; machining without ECA (**a**) and machining with ECA in variant *A* (**b**).

**Figure 8:**
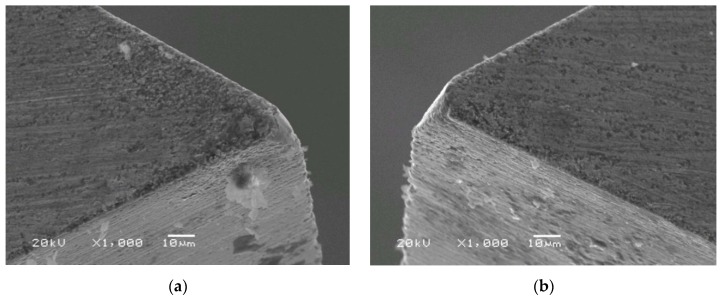
SEM photographs of the cutting edge after removing 10 layers of material with the following cutting parameters: Depth of cut *a_p_* = 1 µm, cutting speed *V_c_* = 80 m/min, feed rate *f* = 3 µm/rev; machining without ECA (**a**) and machining with ECA in variant *B* (**b**).

The SEM (JEOL JSM-5510LV scanning electron microscope, JEOL Ltd., Tokyo, Japan) photographs of the working tool surfaces are presented in Figure 7 and Figure 8. We can conclude that main types of wear in both cases are abrasive and adhesive, and based on the qualitative analysis, it can be summarized that applying ECA has a slight influence on the decreasing of tool tip and main cutting edge wear. However, in variant *A*, the etching effect occurred on the intersection of the main and auxiliary flank. The tool applied during test was made of cermet with a binding phase based on tungsten and cobalt. Therefore, the etching can be explained by electrochemical reactions that take place between tungsten, tungsten carbide, and water, which react to form tungsten trioxide WO_3_, which is then attached to the tool in the form of porous sludge. Due to the charge flow concentration (edge effect), this takes place especially on the intersection of the main and auxiliary flank.

## 4. Conclusions

The current study presents the application of electrochemical assistance in the process of microturning. Depending on the parameters of ECA, material was removed with simultaneous electrochemical and mechanical action (variant *A*) or by application of electrochemical reactions to change the mechanical conditions of the processed material (variant *B*). The objective of choosing straight turning for the experiment was to examine the relation between the processed parameters (cutting depth *a_p_*, cutting speed *V_c_*, and feed rate *f*) on the main cutting force *F* with and without ECA. In both cases, ECA was responsible for decreasing the average cutting force from a few to several millinewtons, which in terms of the force relative value is from few to several percentage points. In variant *A* for the examined cutting parameters’ range, ECA provides benefits for the cutting depth *a_p_* = 1 µm (depth of cut *ΔF* higher value was at the measurement error level). In variant *B*, the experiment was performed for *a_p_* = 1 µm only and the analysis for the examined cutting parameters’ range has proved that benefits of electrochemical assistance are most pronounced for feed rate *f* = 1 µm/rev, and as the feed rate increases, the impact of assistance becomes irrelevant. Another fact worthy of mention is that the application of electrochemical assistance slightly decreases tool tip and main cutting edge wear. However, in variant *A*, the etching effect occurred.

The article described machining of stainless steel which is ductile and for which strain hardening occurs during the process. In the microscale machining, the ratio of depth of cut to cutting edge radius (normally less than 1) requires negative tool rake which increases the ploughing effect. Due to this phenomenon, decohesion (chip formation) is disrupted—the cutting tool slides over the machined surface, thus material is not removed but deformed and strain hardened. The increased force connected to the ploughing effect determines the increase of cutting force. The oxide layer is hard, brittle, and much thinner than regular material, so it is removed more easily when applying lower force.

This paper shows the potential benefits of ECA during cutting. Considering that ECA is mostly beneficial when the depth-of-cut is ≤ 1 µm, it must be stated that such a solution is not very effective. Therefore, it should be utilized at the micropart final stage machining, especially when the cutting depth is in the minimal chip thickness range and the ploughing effect is considered as a significant obstacle in the material removal mechanism. Similarly, it is important that the proposed solution can be applied to a limited range of materials, because in variant *A*, only conductive materials, and in variant *B*, only passivating metals or alloys (i.e., aluminum, titanium alloys, or stainless steel) can be processed. 

## Figures and Tables

**Table 1:** Input and constant machining parameters used during experiments of electrochemically assisted microturning in variants *A* and *B*.

	Variant *A*	Variant *B*
**Input Parameters**		
Cutting Speed, *V_c_*	40−120 m/min	
Feed Rate, *f*	1−5 µm/rev	
Depth of Cut, *a_p_*	1−10 µm	1 µm
**Electrochemical Assistance Parameters**		
Initial Interelectrode Gap Thickness, *S*	0.25 mm	
Electrolyte	1% NaNO_3_ Water Solution	
Interelectrode Voltage, *U*	10 V	3 V
**Other Constant Parameters**		
Workpiece Material	1.4301 Stainless Steel	
Tool	Mitsubishi Turning Insert DCET070200R-SN NX2525	
Shaft Diameter *D*	2.9 mm	
Number of Machined Material Layers, *n*	3	10
